# D-chiro-inositol, an aromatase down-modulator, increases androgens and reduces estrogens in male volunteers: a pilot study

**DOI:** 10.1186/s12610-021-00131-x

**Published:** 2021-06-03

**Authors:** Giovanni Monastra, Mónica Vazquez-Levin, Maria Salome Bezerra Espinola, Gabriele Bilotta, Antonio Simone Laganà, Vittorio Unfer

**Affiliations:** 1 Systems Biology Group Lab, Rome, Italy; 2 Experts Group on Inositols in Basic and Clinical Research (EGOI), Rome, Italy; 3 Instituto de Biología y Medicina Experimental (IBYME, CONICET-FIBYME). Consejo Nacional de Investigaciones Científicas y Técnicas de Argentina (CONICET), Buenos Aires, Argentina; 4 Alma Res Fertility Center, Rome, Italy; 5 Department of Obstetrics and Gynecology, “Filippo Del Ponte” Hospital, University of Insubria, Varese, Italy

**Keywords:** DHEAS, épiandrostérone, estradiol, estrone, glycémie, index HOMA, inhibine B, insulinémie, testostérone, DHEAS, Epiandrosterone, Estradiol, Estrone, Glycemia, HOMA index, Inhibin B, Insulinemia, Testosterone

## Abstract

**Background:**

Androgen deficiency affects men in the adulthood, causing several harmful effects at the reproductive and behavioural levels. Since aromatase is an enzyme that catalyses the conversion of androgens to estrogens, and it is responsible for an adequate balance of both sex hormones in males and females, the administration of molecules acting as down modulators may contribute to restore an abnormal enzymatic activity. A prospective pilot study was carried out to investigate the effect of D-chiro-inositol, a putative aromatase down-modulator, on serum levels of testosterone, estradiol, estrone, dehydroepiandrosterone and epiandrosterone from a group of adult male volunteers. Glucose, insulin, follicle-stimulating hormone, luteinizing hormone, inhibin B, D-chiro-inositol and myo-inositol serum levels were also measured.

**Results:**

Male volunteers were selected according to age and body mass index. Subjects with altered glycemia and/or hormonal status, due to advanced age or abnormal weight, were enrolled in the study. Each of the 10 volunteers enrolled took oral D-chiro-inositol (1 g/day) for 1 month. Serum assays of selected markers were performed at baseline (control) and after treatment. D-chiro-inositol administration was associated to reduced serum levels of estrone (− 85.0%) and estradiol (− 14.4%), and increased serum levels of testosterone (+ 23.4%) and dehydroepiandrosterone (+ 13.8%). In addition, epiandrosterone levels were higher (+39%) after treatment. On the other hand, follicle-stimulating hormone, luteinizing hormone and inhibin B did not change. A trend toward a decrease of glycemia, insulinemia and Homeostatic Model Assessment index was observed after D-chiro-inositol treatment, although differences did not reach statistical significance. D-chiro-inositol treatment did not cause any noticeable adverse effect.

**Conclusions:**

Increased androgens and decreased estrogens seem to confirm that D-chiro-inositol acts as an aromatase down-modulator, but with a still unknown mechanism of action. This pilot study opens up new perspectives of research and therapeutic applications for D-chiro-inositol at different dosages and length of treatment.

Authorization number 005/2020 released by the Local Ethics Committee of Alma Res Fertility Center, Rome.

**Trial registration number:**

NCT04615767 (registry: ClinicalTrials.gov)

Date of registration: November 3, 2020

## Résumé

**Contexte:**

L’insuffisance en androgènes affecte les hommes à l’âge adulte, causant plusieurs effets nocifs aux niveaux reproductif et comportemental. Puisque l’aromatase est. une enzyme qui catalyse la conversion des androgènes en œstrogènes, et qu’elle est. responsable d’un équilibre adéquat des hormones sexuelles chez les hommes et les femmes, l’administration de molécules agissant comme freinateurs peut contribuer à restaurer une activité enzymatique anormale. Une étude prospective pilote a été menée pour étudier l’effet du D-chiro-inositol, un potentiel freinateur de l’aromatase, sur les taux sériques de testostérone, estradiol, estrone, déhydroépiandrostérone et d’épiandrostérone d’un groupe d’hommes adultes volontaires. Le glucose, l’insuline, l’hormone folliculostimulante, l’hormone lutéinisante, l’inhibine B, le D-chiro-inositol et les taux sériques de myo-inositol ont également été mesurés.

**Résultats:**

Les hommes volontaires ont été sélectionnés selon l’âge et l’indice de masse corporelle. Les hommes qui présentaient une glycémie et/ou un statut hormonal altérés en raison d’un âge avancé ou d’un poids anormal, ont été inclus dans l’étude. Chacun des 10 volontaires enrôlés a pris du D-chiro-inositol (1 g/jour) par voie orale pendant un mois. Des dosages sériques des marqueurs sélectionnés ont été réalisés avant (témoin) et après le traitement. L’administration de D-chiro-inositol a été associée à une réduction des taux sériques de l’estrone (− 85.0%) et de l’estradiol (− 14,4%), et a une augmentation des taux sériques de testostérone (+ 23,4%) et de déhydroépiandrostérone (+ 13,8%). En outre, les taux d’épiandrostérone étaient plus élevés (39%) après le traitement. D’autre part, les taux d’hormone folliculostimulante, d’hormone lutéinisante et d’inhibine B n’ont pas été modifiés. Une tendance à la diminution de la glycémie, de l’insulinémie et de l’indice d’évaluation du modèle homéostatique a été observée après traitement par D-chiro-inositol, bien que les différences n’aient pas atteint une signification statistique. Le traitement par D-chiro-inositol n’a causé aucun effet indésirable notable.

**Conclusions:**

L’augmentation des androgènes et la diminution des œstrogènes semblent confirmer que le D-chiro-inositol agit comme un freinateur de l’aromatase, mais avec un mécanisme d’action encore inconnu. Cette étude pilote ouvre de nouvelles perspectives de recherche et d’applications thérapeutiques pour le D-chiro-inositol à différents dosages et durées de traitement.

## Introduction

Testosterone (T) and Estradiol (E2) are essential in male physiology. Both hormones participate to the development of sexual characteristics during adolescence and contribute to general health in the adulthood [1–6]. The biosynthesis of T and its conversion to E2 involve enzymatic processes that maintain the delicate balance between the two hormones. Hence, an adequate regulation of the participating enzymes is of primary importance. In particular, the enzyme aromatase catalyzes the peripheral conversion of androgens to estrogens. Aromatase, also known as estrogen synthetase (gene CYP19A1), is widely expressed in several tissues and organs of the human body, including testis, granulosa of ovarian follicles, placenta, skin fibroblasts, prostate, adipose tissue, bones, breast, brain [7]. Furthermore, it was found also in vascular smooth muscle cells [8], skeletal muscles [9], liver [10] and gastric mucosa [11]. In particular, the adipose tissue that is very rich in aromatase content [12], produces considerable amount of circulating estrogens [13].

Alterations in the aromatase activity lead to an hormonal imbalance. In fact, excessive aromatase activity results in reduced levels of T and increased concentration of estrogens [7, 14].

Men over 30 years old experience 1% annual reduction of serum T [15, 16], with negative impact in their reproductive health. In addition, aromatase-dependent conversion of androstenedione (A4) into estrone (E1) increases with age and obesity [17].

Male androgen deficiency in the adulthood is highly prevalent, with rates increasing with age, from 4% (40–49 years) to nearly 50% (over 80 years). Some studies suggest a 17% increase in deficiency of T for every decade of life [18–20]. Hormonal replacement therapy with T seems associated with possible severe side effects that include cardiovascular and thrombotic events, and polycythemia [21–30]. Therefore, the use of aromatase inhibitors (AIs) has attracted an increasing interest in the last decades [31, 32]. The latest generation of AIs, such as letrozole and anastrozole, inhibit the enzyme activity in a very specific fashion, but they also induce E2 depletion states that may expose patients to osteoporosis and cardiovascular risk [33], In this context, D-chiro-inositol (DCI) may constitute a safer alternative to AIs for increasing androgen levels. DCI is one of the nine inositol stereoisomers, a family of cyclic poly-alcohols that play several key functions in cell physiology [34]. While a small amount of DCI comes from dietary intake, endogenous production provides the majority of human body requirement. Indeed, an insulin-dependent epimerase synthesizes DCI from myo-inositol (Myo-Ins), which is the main component (over 99%) of the cellular inositol pool. Such reaction occurs especially in the liver, muscles and blood, where the highest conversion rate (~ 60%) is reported [35]. As inositol phosphoglycans (IPGs), both MI and DCI are insulin second messengers and influence cell metabolism, by activating key enzymes involved in oxidative and non-oxidative glucose metabolism [36]. Specifically, DCI participates in glycogen synthesis.

In the ovary, DCI modulates insulin-induced androgen biosynthesis [37] and increases T levels either directly, stimulating T biosynthesis in human ovarian thecal cells [38], and indirectly, decreasing the *CYP19A1* aromatase gene expression in granulosa cells [39]. Based on these premises, researchers suggested that treatment with high doses of DCI may have clinical application in conditions where decreased estrogen levels is required [40, 41].

The aim of this pilot study was to test the effect of a 30-day DCI treatment on, upon T, E2, T/E2 ratio, Dehydroepiandrosterone sulfate (DHEAS), E1, Homeostatic Model Assessment (HOMA) index, glycemia, insulinemia, Follicle-stimulating hormone (FSH), Luteinizing hormone (LH), epiandrosterone (Epia) and inhibin B levels in male volunteers (V) selected by their age and body mass index (BMI). Serum concentrations of DCI and MI were also determined before and after DCI supplementation.

## Patients and methods

### Subject eligibility

This prospective study was approved by the Local Ethics Committee of Alma Res Fertility Center in Rome, Italy (registration n. 005/2020), where the trial was carried out. Enrolment involved only male volunteers, evaluated on the basis of medical history, physical examination, and laboratory hormone screening (details below). All patients provided a written informed consent to participate in the study. Eligibility criteria were the following: (1) age range 30–65 years; (2) BMI between 22 and 34 kg/m^2^; (3) moderate alteration of glycemia and/or testosterone and estradiol levels (within 15% of the normal range). Individuals with a diagnosed or suspected disease, as well as those regularly taking psychoactive substances, dietary supplements or drugs that may interfere with the endpoints of this study were excluded. All participants were advised to follow their usual diet during DCI treatment.

To assess whether the sample was statistically adequate, a post hoc analysis was performed using the Wilcoxon signed-rank Test for matched pairs, with alpha = 0.05.

### Study design and intervention

All volunteers were instructed to take oral DCI (1.0 g in total) two times a day, morning and evening, on an empty stomach to maximize intestinal absorption. Treatment duration was 30 days. DCI was provided by Amicogen, Gyeongsangnam-do, South Korea in 500 mg capsules (purity of 96.53%) specifically manufactured for this study. Baseline (t0) values were obtained from the volunteers and used as reference.

Given that current information prevents to set exact boundaries to DCI therapeutic profile, dosage and treatment length for this study were determined based on the available evidence on DCI efficacy and safety in males and females [42–48]. Because increased testosterone level is expected after DCI treatment, clinical studies on testosterone administration were also taken into consideration. Indeed, excess T may induce testicular atrophy and sterility due to decreased systemic FSH and LH levels [49]. As DCI leads to a down-regulation of the aromatase enzyme [39], a long-term administration may cause potentially harmful testosterone levels. Considering that all volunteers were basically healthy individuals, a 30-day treatment was deemed adequate to observe hormonal changes without causing imbalance of sex hormones.

The primary outcome of this study was the increase of T/E2 ratio, a reliable index of aromatase activity [50]. Secondary outcomes were changes of T, DHEAS, E1, E2, HOMA index, glycemia, insulinemia, FSH, LH, inhibin B and epiandrosterone. In addition, serum concentrations of DCI and MI were assessed before and after 30 days of supplementation with DCI.

### Sample collection and preparation

Blood samples were collected by venipuncture at the baseline and after 30 days of DCI treatment. Blood samples were centrifuged, and serum was stored at − 20 °C until assayed.

### Serum assays

Serum levels of T, DHEAS, E1, E2, FSH, LH, glycemia, insulinemia, inhibin B and Epia were measured by Raphael Medical Lab, using commercial kits (T: Chemiluminescent Immunoassay System, Beckman Coulter, Brea, CA, USA; DHEAS: Chemiluminescent Immunoassay System, Siemens, Munich, Germany; E1: ELISA kit, Dasit, Milan, Italy; E2: Chemiluminescent Immunoassay System, Beckman Coulter, Brea, CA, USA; Epia: Enzyme Immunoassay Kit, Arbor Assays, Ann Arbor, Michigan, USA; FSH: Chemiluminescent Immunoassay System, Beckman Coulter, Brea, CA, USA; inhibin B: ELISA kit, Skylab, Ansh Labs, Webster, Texas, USA; LH: Chemiluminescent Immunoassay System, Beckman Coulter, Brea, CA, USA; glycemia: Trinder glucose oxidase method, Werfen Company, Milan, Italy; Insulinemia: Chemiluminescent Immunoassay System, Beckman Coulter, Brea, CA, USA). The following standard ranges were used as reference for the analyses: T: 1.75–7.81 ng/ml; E2: 20–47 pg/ml; E1: 9.0–79.1 pg/ml; DHEAS: 80–560 μ/dl; glycemia 70–105 mg/dL; insulinemia 1.9–23.0 μUI/ml; FSH: 1.27–19.26 mUI/ml; LH: 1.24–8.62 mUI/ml; inhibin B: 24–310 pg/ml. Quantification of serum levels of DCI and MI (μmol/l) was performed by Mérieux NutriSciences Italia (Resana–Treviso, Italy), and carried out according to the following procedure. After extraction with organic solvents and derivatization, samples were analyzed by gas chromatography - mass spectrometry with GC/MS Agilent 6890 (Agilent, Santa Clara, CA, USA). The injection (1.0 μl) was performed in a splitless mode at 270 °C, using a capillary column Agilent 122–5532 dB-5 ms (0.25 mm × 30 m × 0.25 μm). The total run-time lasted 15 min: oven at 70 °C from 0 to 1 min; 20 °C/min to 150 °C; 10 °C/ min to 240 °C; 4 min at 320 °C post-run. The flow rate was kept at 1.2 ml/min, and analytes were detected with a 5973 Network Series Mass Selective detector in selected ion monitoring (SIM) modality. Cmax (maximum concentration recorded) and Tmax (time to reach Cmax) were calculated from the chromatograms.

### Statistical analysis

Descriptive statistics summarizing quantitative variables included median values, as well as the 25th and the 75th percentiles. Wilcoxon signed rank sum test was performed to compare changes from t0 (baseline) to t1 (after DCI treatment) for all parameters evaluated in the study. Data are presented using box plots. Statistical analysis was implemented as two-sided testing with 0.05 significance level, using SAS® version 9.4 (SAS Institute Inc. 100 SAS Campus Drive Cary, NC, USA) and Stata™ version 8.2 (StataCorp LLC, College Station, TX, USA).

## Results

Among 42 screened volunteers, ten (V1-V10) satisfied the criteria and entered the study. The post hoc analysis demonstrated that the sample size was statistically adequate, with power of the study greater than 90%.

It is important to point out that none of the study participants reported any type of adverse events.

The age range of the selected volunteers was 30–55 years, with a median value of 37.0 years (25th and 75th percentiles: 34.3 and 42.3, respectively). The BMI median value was 27.5 (26.1 and 29.6). Two subjects had normal weight (BMI: 23.4 and 22.9), 6 were overweight (BMI between 25.8 and 29.7), and 2 were obese (BMI: 30.9 and 33.6). These values were found unchanged after treatment with DCI.

Median, as well as the 25th and the 75th percentile values were determined for T, E2, E1, DHEAS, FSH, LH, inhibin B, glycemia, insulin and HOMA index at the baseline (t0) and after DCI treatment (t1). Table 1 reports the baseline values of glycemia, insulinemia, HOMA index, DCI, MI and BMI for each volunteer.

**Table 1:** Glycemia, insulinemia, Homeostatic Model Assessment (HOMA) index, D-chiro-inositol (DCI), myo-inositol (MI) and Body Mass Index (BMI) values at baseline in volunteers V1-V10

Volunteers	glycemia mg/dl	insulinemia μIU/ml	HOMA index (^a^)	DCI μmol/l	MI μmol/l	BMI
V1	117	32.16	**9.3**	0.15	13.0	29.7
V2	101	4.58	**1.15**	0.16	13.5	27.3
V3	109	5.60	**1.5**	0.16	14.3	29.4
V4	110	4.18	**1.14**	0.25	15.0	27.0
V5	88	6.91	**1.5**	0.29	16.4	23.4
V6	86	9.66	**2.06**	0.49	18.0	27.6
V7	95	8.93	**2.09**	0.70	18.0	22.9
V8	93	8.70	**2**	0.71	20.8	25.8
V9	110	7.19	**1.96**	0.90	20.8	33.6
V10	90	5.82	**1.29**	0.90	21.0	30.9
**Percentiles**						
25	90.8	5.7	1.3	0.18	14.5	26.1
**50**	**98.0**	**7.1**	**1.5**	0.39	17.2	**27.5**
75	109.8	8.9	2.1	0.71	20.8	29.6

Individual results and Percentiles (25th, 50th and 75th) of Glycemia, Insulinemia, *BMI* Body Mass Index, *HOMA* Homeostatic Model Assessment, *DCI* D-chiro-inositol and *MI* myo-inositol in the Study cohort of 10 volunteers (V1-V10)

(^a^) arbitrary units

All men had normal baseline levels of T (median: 4.4; 25th and the 75th percentile values: 3.9 and 4.6, respectively), E2 (median: 27.4 percentiles: 25.3 and 30.3), T/E2 (median: 16.8; percentiles: 13.3 and 19.0), FSH (median: 8.2; percentiles: 4.8 and 10.7), LH (median: 3.8; percentiles: 3.2 and 4.4) and inhibin B (median: 110.4; percentiles: 97.7 and 126.7). Conversely, six volunteers exhibited higher E1 levels than normal (109.4, 121.6, 103.0, 166.6, 105.2, 260.0 pg/ml); E1 median value at baseline was 104.1 pg/ml (percentiles: 63.9 and 118.6). These anomalies were unrelated to age or BMI (data not shown). Three subjects had baseline DHEAS levels lower than normal (66.5, 22.0, 54.0 μg/dl), but they increased after DCI treatment; the other volunteers had baseline DHEAS levels within the physiological range (80–560 μg/dl); DHEAS median value at baseline was 210.0 μg/dl (percentiles: 73.9 and 282.8).

One obese (V9) and three overweight subjects (V1, V3, and V4) had moderately higher glycemia than normal. In contrast, one obese (V10) and three overweight volunteers (V2, V6, and V8) were normoglycemic.

Overall, the evaluated sample group displayed the variability found in a western population, with a prevalence of overweight subjects.

**Fig. 1:**
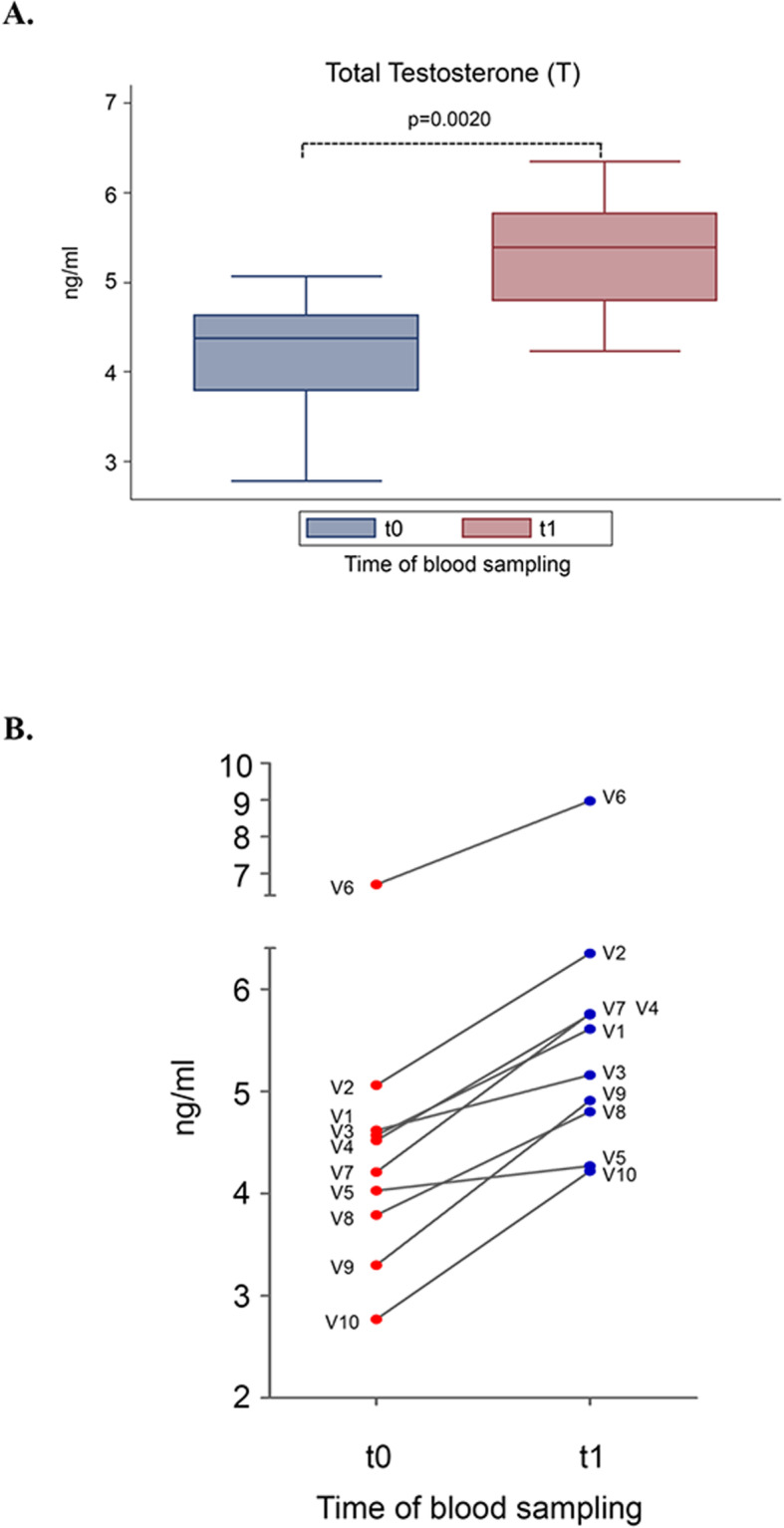
Effect of D-chiro-inositol (DCI) treatment on total testosterone (T) levels. Total serum T levels from 10 male volunteers at baseline (t0) and after 30 days of treatment (t1) with 1 g DCI per day are presented as: **a** median values of total T (box plots). The boxes denote the interquartile range between the first and third quartiles (25th and 75th percentiles, respectively), the whiskers represent the minimum and maximum values, and the line inside the boxes represents the median value (*p* = 0.0020; Wilcoxon signed rank sum test); **b** individual total T values (before-after treatment). The graph reports the values for each volunteer participating in the study

DCI administration for 30 days improved all sex hormone values analyzed in this pilot study. In particular, DCI treatment resulted in a 23.4% increase of T (*p* = 0.0020) (Fig. 1a). This remarkable effect was observed in nine out of 10 volunteers, even if baseline T levels were already withing the normal range (Fig. 1b).

**Fig. 2:**
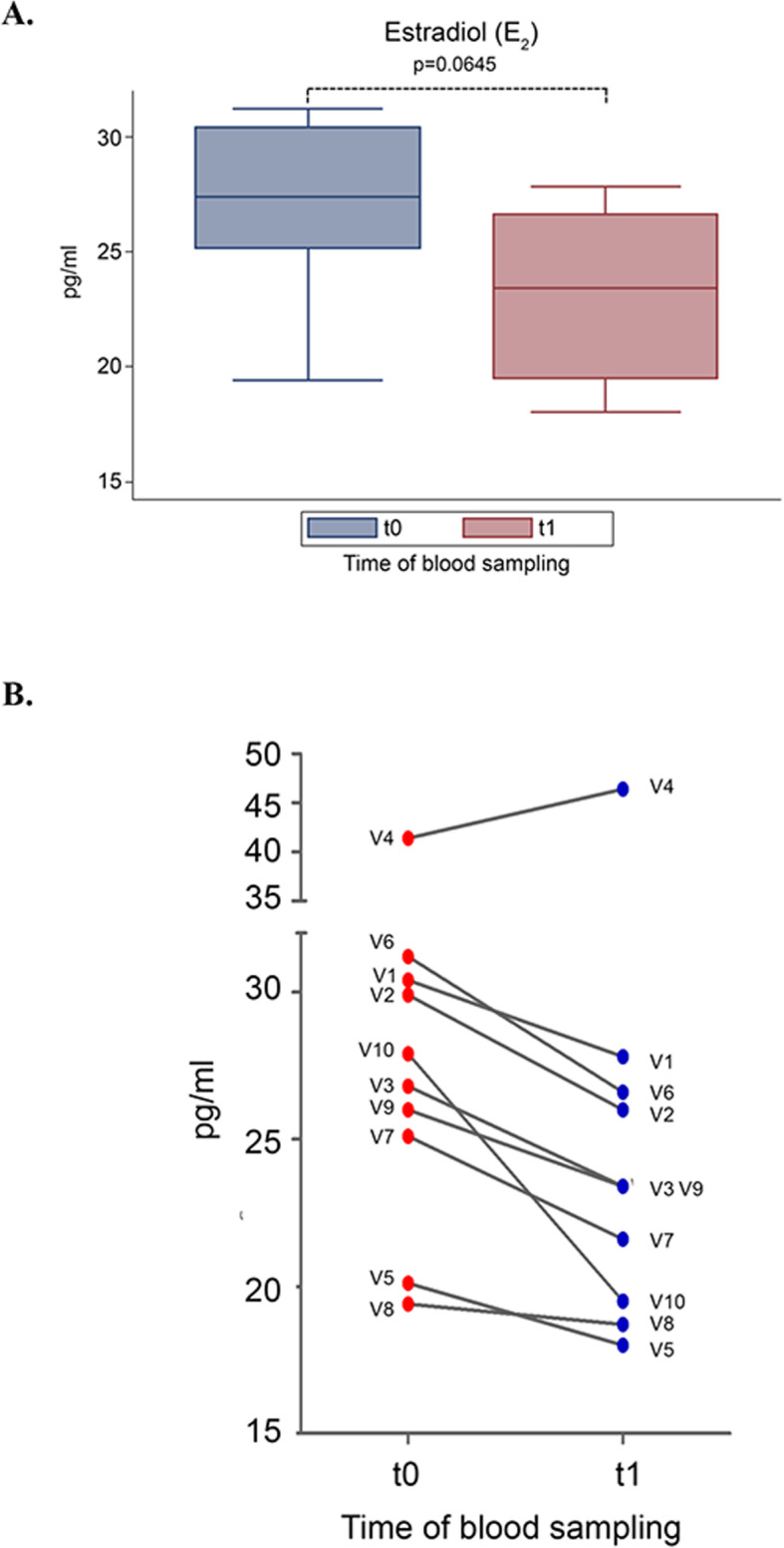
Effect of D-chiro-inositol (DCI) treatment on Estradiol (E2) levels**.** E2 levels from 10 male volunteers at baseline (t0) and after 30 days of treatment (t1) with 1 g DCI per day are presented as: **a** median values of E2 (box plots). (*p* = 0.0645; Wilcoxon signed rank sum test). Symbol explanation: see caption of Fig. 1a; **b** individual E2 values (before-after treatment). The graph reports the values for each volunteer participating in the study

Moreover, DCI treatment induced an overall 14.4% decrease of E2 values. Although such decrease was observed in 9 out of 10 individuals, the differences failed to reach statistical significancy (*p* = 0.0645) (Fig. 2a). Only one volunteer experienced increased E2 levels at t1 (V4, t0 = 41.4 pg/ml; t1 = 46.4). (Fig. 2b).

**Fig. 3:**
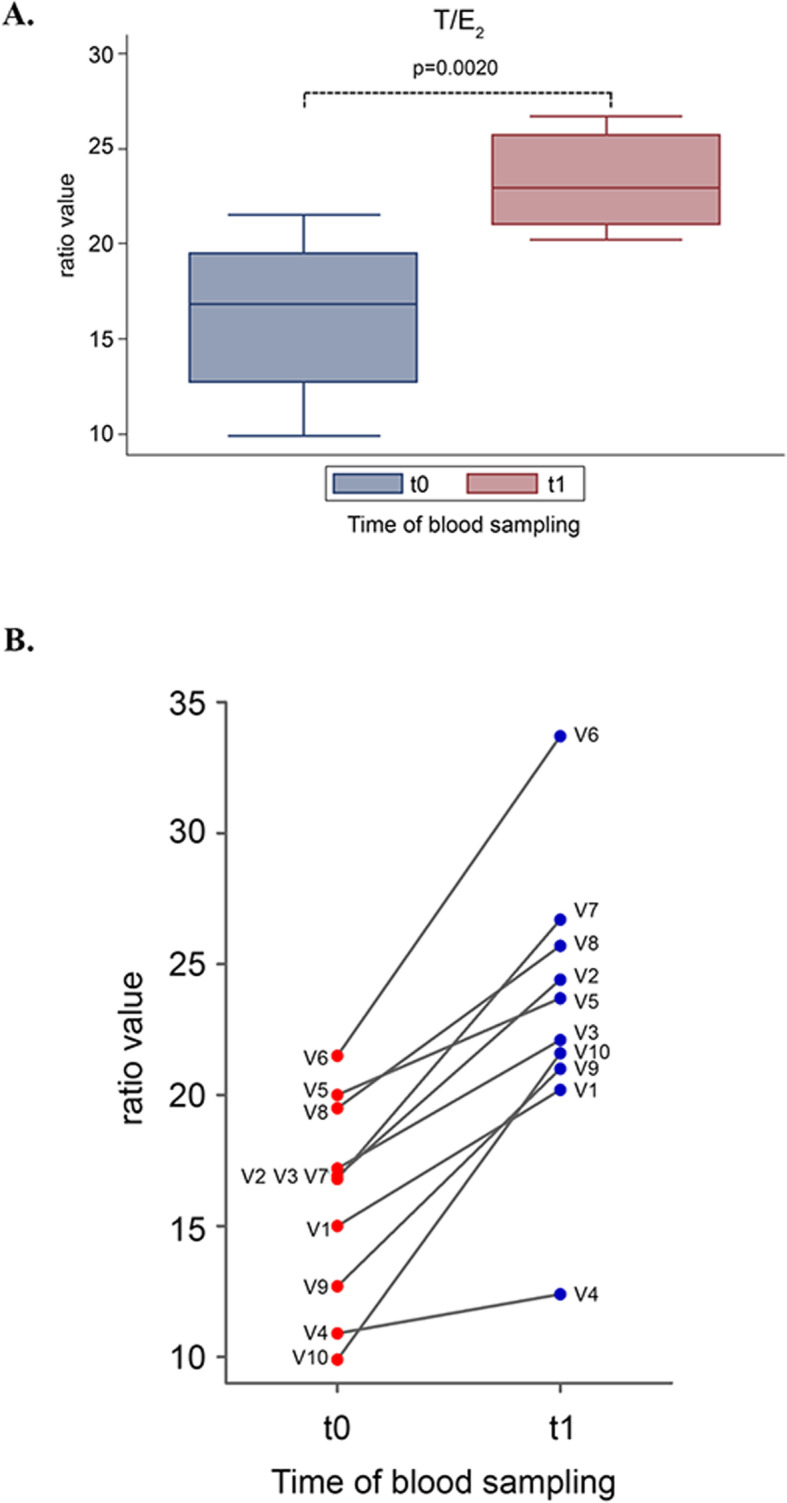
Effect of D-chiro-inositol (DCI) treatment on testosterone/estradiol (T/E2) ratio. T/E2 ratio from 10 male volunteers at baseline (t0) and after 30 days of treatment (t1) with 1 g DCI per day are presented as: **a** median values of T/E2 ratio (box plots) (*p* = 0.0020; Wilcoxon signed rank sum test). Symbol explanation: see caption of Fig. 1a; **b** individual T/E2 ratio (before-after treatment). The graph reports the values for each volunteer participating in the study

**Fig. 4:**
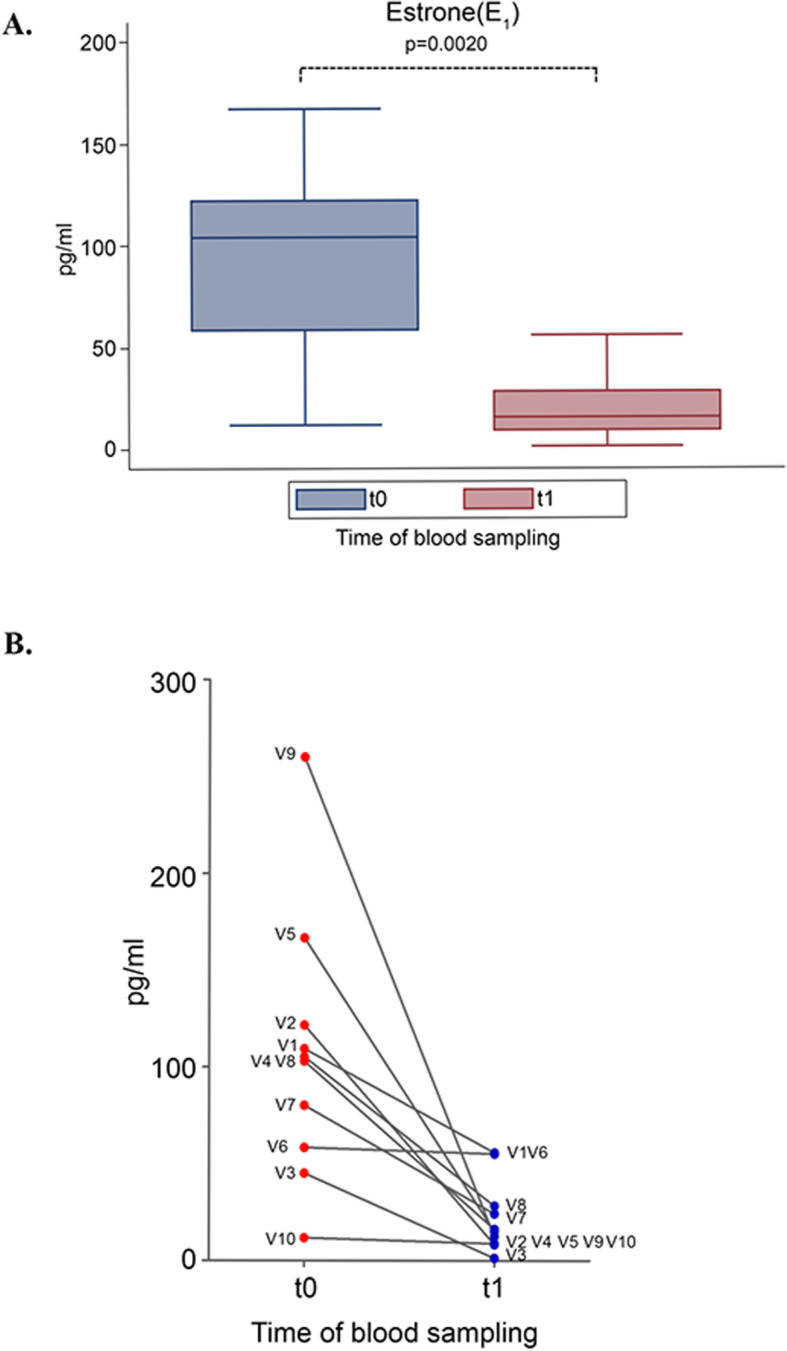
Effect of D-chiro-inositol (DCI) treatment on estrone (E1) levels. E1 levels from 10 male volunteers at baseline (t0) and after 30 days of treatment (t1) with 1 g DCI per day are presented as: **a** median values of E1 (box plots) (p = 0.0020; Wilcoxon signed rank sum test). Symbol explanation: see caption of Fig. 1a; **b** individual E1 levels (before-after treatment). The graph reports the values for each volunteer participating in the study

Based on these results, DCI treatment significantly increased the overall T/E2 ratio by 36% (*p* = 0.0020) (Fig. 3a). The individual results of T/E2 ratios for each patient revealed a consistent increase, with a pattern very similar to T (median values of T/E2 ratio, t0 = 10.9%, t1 = 12.3%) (Fig. 3b):

In addition to these findings, DCI treatment resulted in an overall 85% decrease of E1 values when compared to those at baseline (Fig. 4a). All the subjects with baseline E1 levels above the physiological range exhibited normal values after DCI treatment (Fig. 4b). Indeed, E1 decreases in all subject except one (V6), who maintained almost unaffected E1 levels. However, DCI effect on the increase of T levels in this case was remarkable (+ 33.9%).

**Fig. 5:**
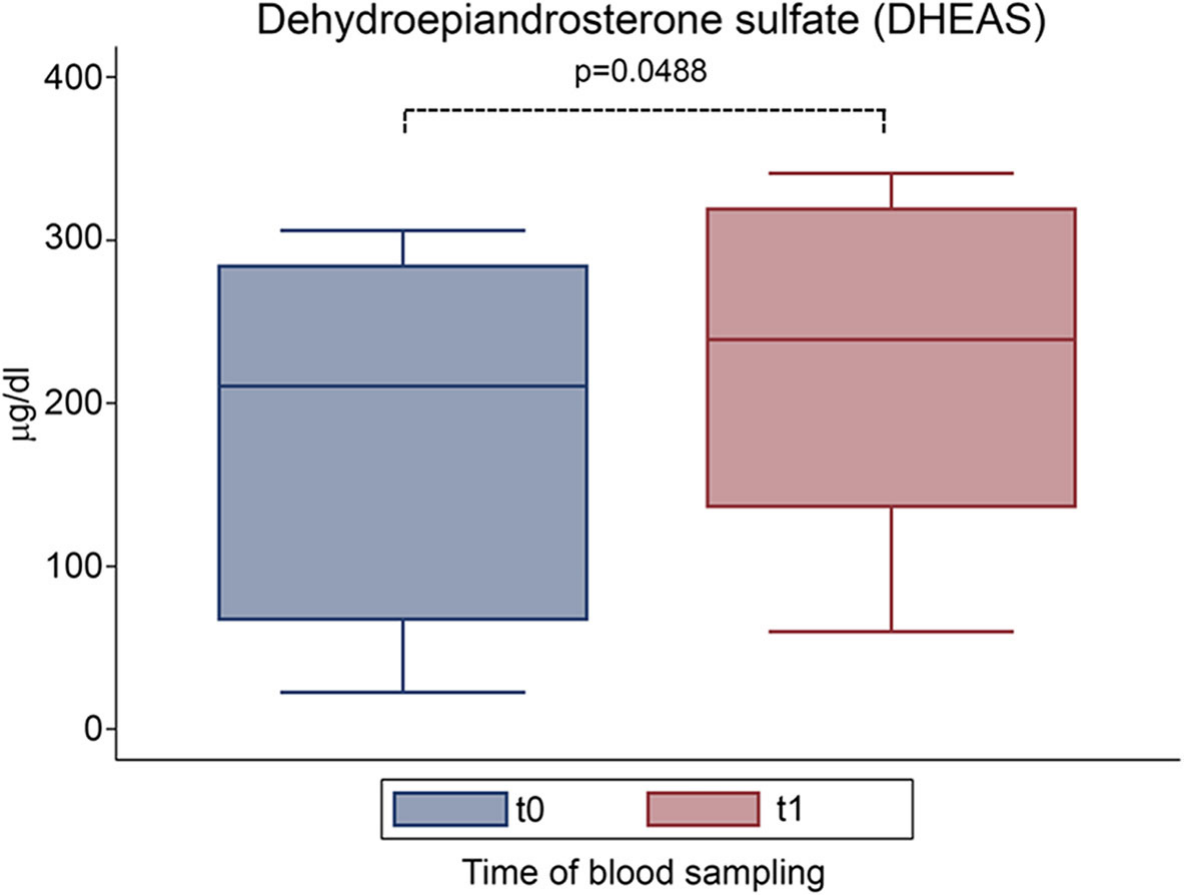
Effect of D-chiro-inositol (DCI) treatment on dehydroepiandrosterone sulfate (DHEAS) levels. DHEAS levels from 10 male volunteers at baseline (t0) and after 30 days of treatment (t1) with 1 g DCI per day are presented as median values of DHEAS (box plots) (*p* = 0.0488; Wilcoxon signed rank sum test). Symbol explanation: see caption of Fig. 1a

Moreover, the overall DHEAS concentration increased by 13.8% after DC1 treatment (t0 = 210 μg/dl, t1 = 239 μg/dl; *p* = 0.049). Both T and DHEAS remained within the normal range after the treatment (Fig. 5).

**Fig. 6:**
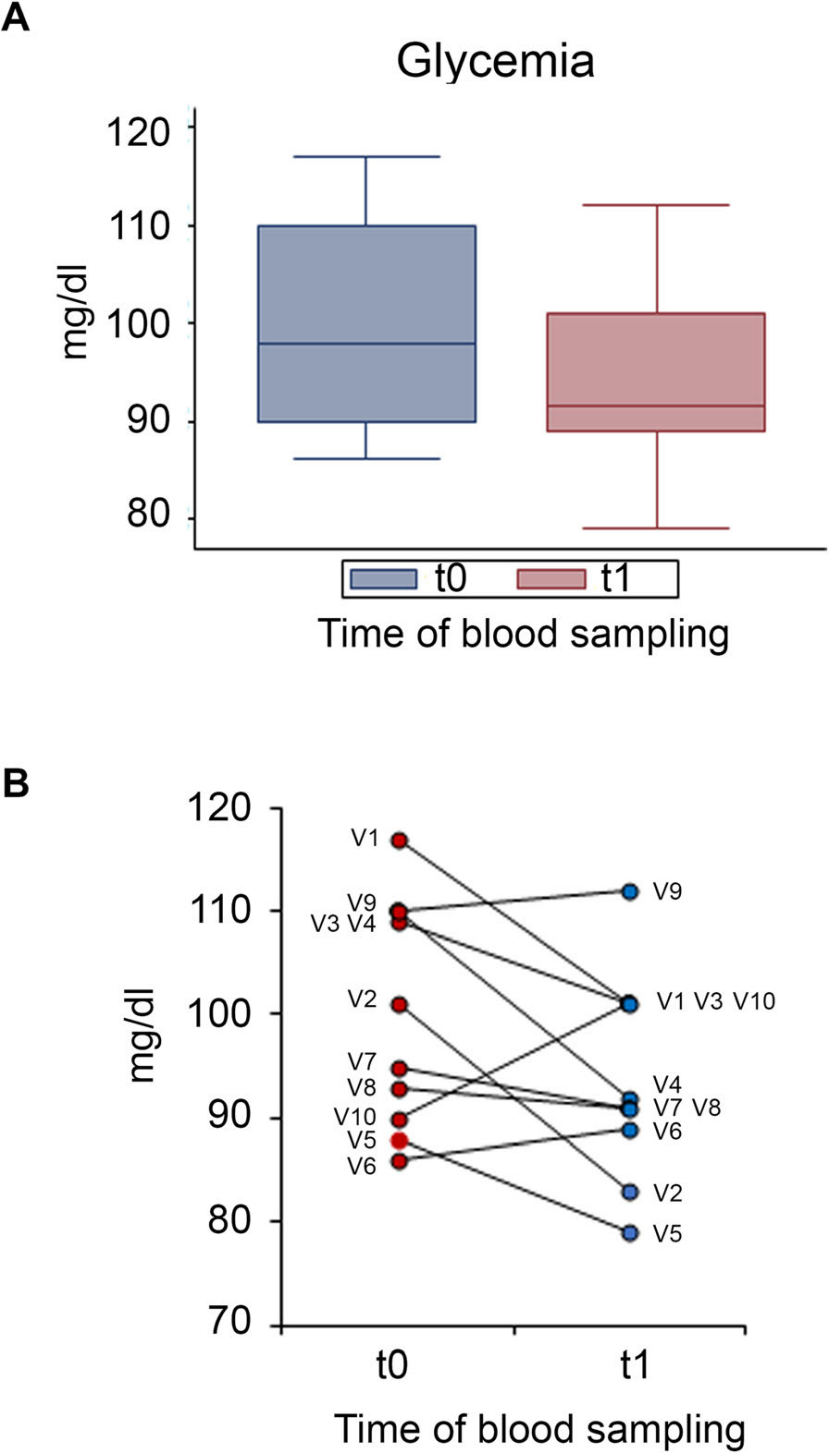
Effect of D-chiro-inositol (DCI) treatment on glycemia levels. Glycemia levels from 10 male volunteers at baseline (t0) and after 30 days of treatment (t1) with 1 g DCI per day are presented as: **a** median values of glycemia (box plots) (*p* = 0.1113; Wilcoxon signed rank sum test). Symbol explanation: see caption of Fig. 1a; **b** individual glycemia levels (before-after treatment). The graph reports the values for each volunteer participating in the study

**Fig. 7:**
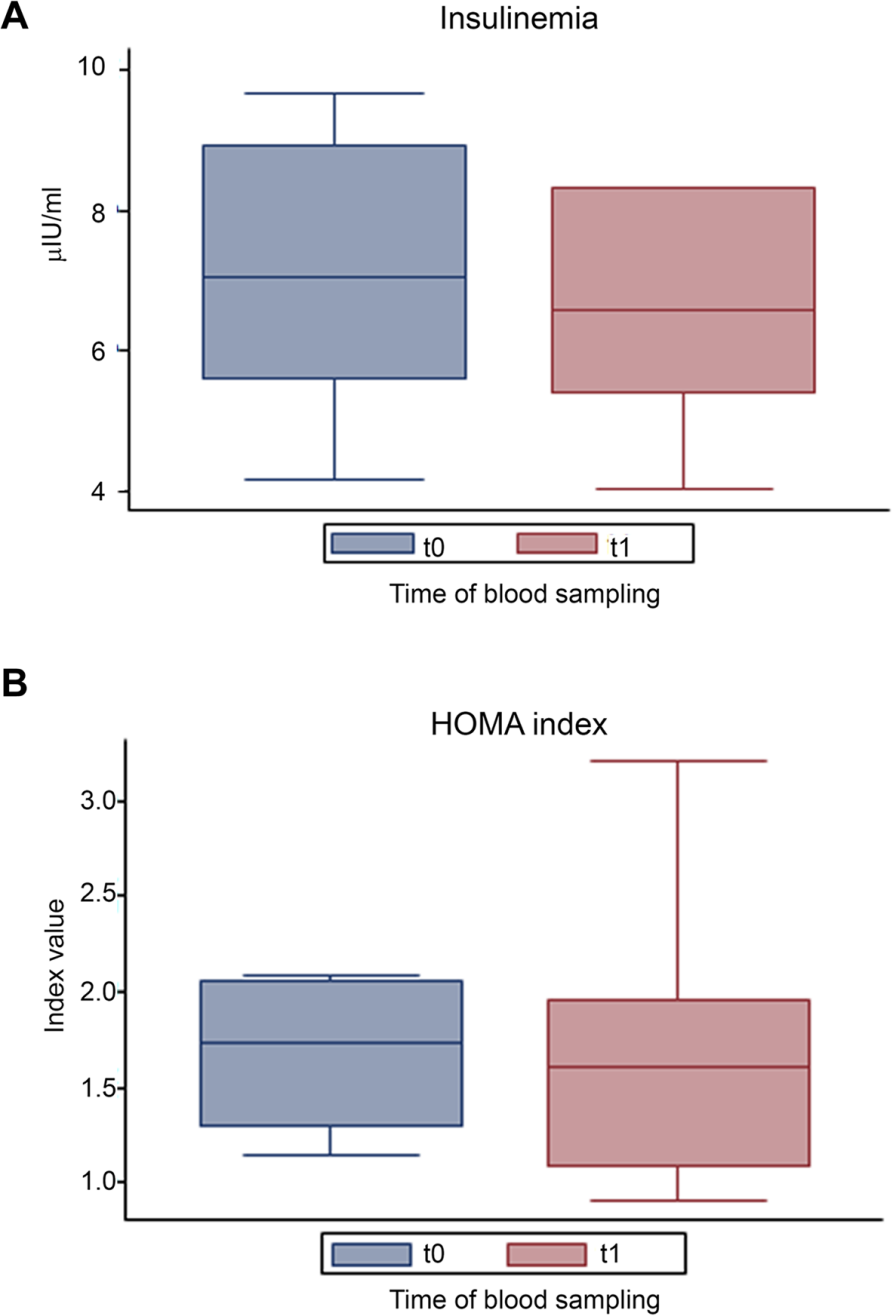
Effect of D-chiro-inositol (DCI) treatment on insulinemia and HOMA index levels. Insulinemia and HOMA index from 10 male volunteers at baseline (t0) and after 30 days of treatment (t1) with 1 g DCI per day are presented as median values of (**a**) insulinemia and (**b**) HOMA index (box plots) (insulinemia, *p* = 0.3750; HOMA index, *p* = 0.3594; Wilcoxon signed rank sum test). Symbol explanation: see caption of Fig. 1a

**Fig. 8:**
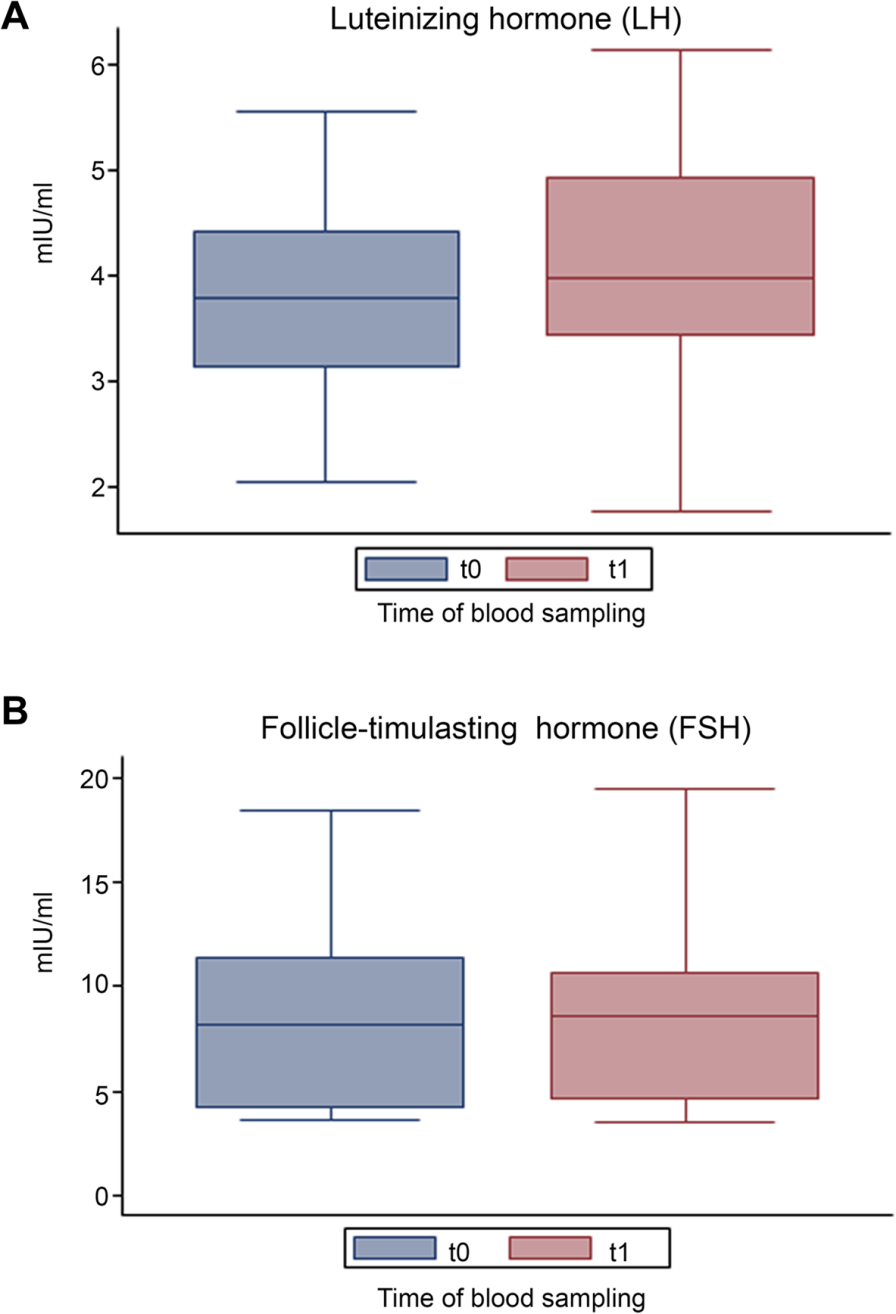
Effect of D-chiro-inositol (DCI) treatment on Luteinizing hormone (LH) and follicle-stimulating hormone (FSH) levels. LH and FSH levels from 10 male volunteers at baseline (t0) and after 30 days of treatment (t1) with 1 g DCI per day are presented as median values of (**a**) LH and (**b**) FSH levels (box plots) (LH, *p* = 0.3340; FSH, *p* = 0.5566; Wilcoxon signed rank sum test). Symbol explanation: see caption of Fig. 1a

Glycemia, insulinemia and HOMA index were also evaluated before and after DCI treatment. While no significant differences were found between t0 and t1, a trend towards a decrease was observed for all three outcomes (Figs. 6 and 7). DCI treatment restored glycemia to normal values in 3 out of 4 males with abnormal baseline levels (the individual values decreased from 117, 109 and 110 to 101, 101 and 92, respectively). No significant changes were found for LH and FSH serum levels after DCI treatment, compared to the baseline. (Fig. 8).

Inhibin B levels were also determined in all 10 volunteers, finding no changes when comparing results before and after DCI administration. The median value was 110.4 pg/ml (percentiles: 97.7 and 126.7) at baseline, and 110.7 pg/ml (percentiles: 99.0 and 126.2) at t1.

After obtaining the data described above, Epia serum levels were also assessed. As a sufficient sample amount was not available from all samples to run individually, a preliminary evaluation was carried out on pooled sera from all ten volunteers. The results revealed a 39% increase in Epia concentration, with a baseline value of 44.62 ng/ml and a t1 value of 62.14 ng/ml.

### Pharmacokinetics of DCI single dose

**Fig. 9:**
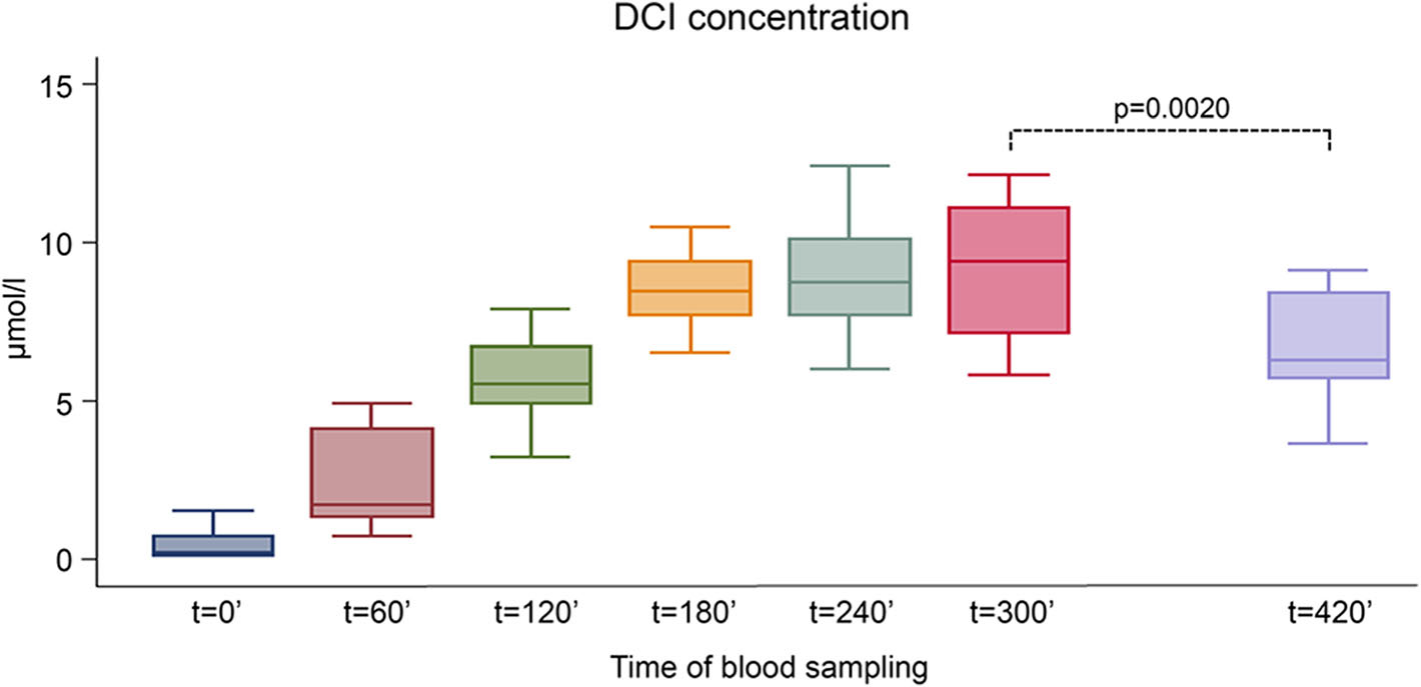
D-chiro-inositol (DCI) pharmacokinetics. DCI concentration (μmol/l) in serum from 10 male volunteers at different time points after oral administration of 1 g DCI (single dose). Median values of DCI at different time points after oral administration are reported (Cmax: 9.40 μmol/l, Tmax: 240 min, AUC (0–420): 2750.59). After 420 min, DCI concentration is significantly decreased in comparison to 300 min (p = 0.0020). Symbol explanation: see caption of Fig. 1a

To evaluate the pharmacokinetic profile of DCI in the study participants, all volunteers ingested a single dose (1 g) of DCI ten days before starting the treatment for the study. The results are shown in Fig. 9. After a single DCI administration, serum levels peaked at 240 min, remaining almost constant for one additional hour. The values of the main parameters were: Cmax: 9.40 μmol/l; Tmax: 240 min; AUC (0–420): 2750.59. The average serum concentration increased forty-seven-fold with respect to baseline values (median value: 0.2 μmol/l). These results were also compared with previous similar studies carried out with MI. Of note, the shape of the DCI curve is shifted to the right in comparison with MI, which showed a peak at 180 min [51]. This result led us to suggest that DCI has a longer serum half-life than MI. At 420 min, DCI levels significantly decreased when compared to those determined at 300 min (*p* = 0.0020).

### DCI and MI serum levels before and after DCI treatment

DCI and MI serum concentrations were within the normal range in all volunteers before starting the study treatment (0.18–0.40 μmol/l for DCI, and 15–30 μmol/l for MI). Accordingly, the MI/DCI ratio was very close to physiological values (40:1 in serum). After the treatment, DCI levels increased about 40-fold (*p* = 0.0001), while the MI concentration had a much smaller change (+ 30%), as reported in Table 2.

**Table 2:** D-chiro-inositol (DCI) and myo-inositol (MI) in blood at baseline and after 30 days DCI treatment in volunteers V1-V10

Time	Percentile	DCI μmol/l	MI μmol/l	MI/DCI
t0	25	0.18	14.5	44:1
	**50**	**0.39**	**17.2**	
	75	0.71	20.8	
t1	25	12.5	18.4	1.5:1
	**50**	**15.2**	**22.5**	
	75	18.7	25.9	

Results of *DCI* D-chiro-inositol, *MI* myo-inositol and MI/DCI ratio at baseline (t0) and after DCI treatment (t1) expressed as Percentiles (25, 50 and 75) for 10 volunteers (V1-V10)

## Discussion

This pilot study was carried out in male volunteers, mainly constituted by overweight individuals. Patient treatment with DCI reduced serum concentrations of estrogens E2 and E1, with a stronger effect on the latter. Moreover, DCI administration resulted in increased levels of androgens T and DHEAS, both remaining within the normal range. Based on these findings, an improvement in T/E2 ratio was achieved at the end of the treatment. DCI restored normal values of glycemia in almost all subjects with levels above the physiological range, however such changes did not reach statistical significancy. Longer periods of administration may be required to observe anti-glycemic effects in all patients. FSH, LH and inhibin B levels of all 10 volunteers, all within the normal range at t0, were found unchanged after DCI treatment.

Taking into account the values of glycemia for each participant and the lack of significancy for variations related to the treatment, DCI-induced changes of sex hormone balance seem unrelated to any previous modification of glycemia.

In the present study, no significant changes were found in inhibin B serum levels after DCI treatment. Previously, Calogero et al. [52] reported an increase in inhibin B levels following MI treatment (4 g per day for 3 months) in men with idiopathic infertility. The patients included were below 45 years, with age and BMI average values of 28 ± 9 years and 26.6 ± 2.7, respectively. The authors found a significant decrease of LH and FSH, and an increase of inhibin. Beside the use of different inositol isomers, other distinctions between such study and ours must be pointed out. In the study of Calogero et al., MI dosage was four-fold higher than DCI in our study, and the treatment period was three times longer. Furthermore, the characteristics of the study population are quite different between the two studies (age, BMI, etc.). Calogero et al. [52] found an average baseline inhibin B value of 86 ± 24 pg/ml. Although within the normal range (24–310 pg/ml in the overall population) [53], the reported values were lower than those found in our population (110.4 pg/ml at t0 and 110,7 pg/ml at t1).

We think that DCI exerts very specific effects and the evidence available so far does not allow to employ it in the therapy for male infertility, where MI is the only inositol isomer to be used for the treatment.

Other two studies from the same research group report the impact of DCI in male reproduction and health. The first one evaluated the effects of DCI on spermatozoa in vitro [54], using semen samples from 15 asthenozoospermic patients and 15 normozoospermic healthy males. Samples were incubated with different DCI concentrations (0, 75, and 750 μg/mL) for 30 min, resulting in increased sperm mitochondrial membrane potential (MMP), thus demonstrating for the first time a positive in vitro effect of DCI on sperm function of asthenozoospermic patients. However, previous similar experiments with MI gave better results, as MI improved the abnormal sperm parameters in males with oligo-astheno-teratozoospermia (OAT), besides those in asthenozoospermic subjects. Of note, MI exerted its effects in the same incubation time used for DCI (30 min.) [55]. Furthermore, Condorelli et al. did not evaluate progressive motility, whereas it was significantly improved by in vitro MI treatment [55].

The second study [56] assessed the effect of estrogen modulation on prostatic health, as hyperestrogenism is associated with prostate inflammation. These data are convincing and open therapeutic perspectives for the use of DCI, that could help maintain prostate health. However, in our opinion, the best target in males for DCI administration is “mild hypogonadism”.

As mentioned, DCI treatment in healthy volunteers resulted in higher levels of DHEAS, from which Epia derives. However, DHEAS increased by 13.8%, whereas Epia by 39%. Further studies are necessary to confirm these differences and their significance. Although our data refer only to male patients, it is interesting to highlight that high levels of Epia are associated with a substantial risk of developing PCOS in females [57]. Such increase can be considered a risk indicator, should the same trend be confirmed in women treated with DCI. In-depth specific studies should be carried out in the future to better understand this intriguingly complex set of events.

Based on the observed increased androgens and decreased estrogens, we are starting to consider DCI as an aromatase modulator, although the precise mechanism (direct or indirect) of action remains to be elucidated. The aromatase modulation is a dose-dependent phenomenon, and different tissues and organs respond in a different manner. This may also explain the partial efficacy of aromatase inhibitors in men [31]. An incomplete suppression may be regarded as advantageous because it prevents excessive reduction of estrogen levels in men and the possible associated adverse effects [31]. This is a research area that may be addressed in future studies to investigate in depth the activity of DCI as an aromatase down-regulator.

In our study, DCI treatment modified E1 serum levels in a remarkable way. At baseline, its median value was above the range, as 6 volunteers had abnormal levels. This value became normal after DCI treatment, and in all six men E1 levels were restored to normal. A study published in 2013 demonstrated that E1 is a reliable predictive marker of Type 2 Diabetes Mellitus (T2DM) [58]. In the report of a community-dwelling men, the authors found that E1 levels were prospectively associated with incidental T2DM. Conversely, analogous association was not found with E2. The significant association between E1 and the risk of T2DM could be explained by the differential actions of E1 on estrogen receptor subtype alpha (Erα) and estrogen receptor subtype beta (Erβ) [59, 60]. E1 and E2 bind to both Erα and Erβ, with E2 exhibiting greater affinity and activity than E1 in several in vitro tests. The associated specificity can lead to different pharmacological effects of estrogen receptor modulators. For example, randomized trials revealed an increased risk of T2DM in women treated with tamoxifen, compared to those from a placebo group. Instead, raloxifen treatment was not associated with analogous risk [61]. Accordingly, the correlation of E1, but not E2, with T2DM risk may be ascribable to their differential activity on estrogen receptor subtypes [58]. The biologic function played by E1 in men is often under-considered. Although E1 binds to receptors with lower affinity than E2 in some bioassays, circulating E1 levels in men are often appreciable [58]. Moreover, E1 can be converted to E2 in the organism. Furthermore, whether E1 exerts additional non-genomic effects on insulin secretion or sensitivity is still unknown, therefore the signal pathways involved in the E1 role on the T2DM risk should be explored. In the present study, we found that DCI reduces E1 levels, suggesting that DCI counteracts the onset of T2DM not only through its direct hypoglycemic activity related to its involvement in insulin signalling pathway [62, 63]. DCI activates glycogen synthase and stimulates pyruvate dehydrogenase phosphatase, supporting ATP synthesis and favouring overall glucose cellular management [62]. Based on this profile, DCI alone was suggested for treating hyper-insulinemic illnesses such as metabolic syndrome, T2DM and Polycystic Ovary Syndrome (PCOS) [42–48], although some notable concerns have arisen in recent years about its use in PCOS [64–66].

DCI activity as hypoglycemic agent was recently confirmed by a preclinical study [67] that highlighted a new mechanism of action of DCI to decrease gluconeogenesis in insulin-resistant hepatocytes. This effect occurs via PI3K/AKT/FOXO1-mediated inhibition of glucose-6-phosphatase and cytosolic phosphoenolpyruvate carboxykinase mRNA expression [67]. In the study, DCI was able to reduce hepatic gluconeogenesis and endogenous glucose consumption, not only in human HepG2 cells but also in insulin-resistant mice. The decreased gluconeogenesis accounts for DCI-induced lowering of blood glucose levels and amelioration of glucose homeostasis and insulin resistance (IR) [67]. Such finding increased the number of different mechanisms through which DCI counteracts peripheral IR, observed also in other experimental models where IR was induced by glucosamine [68]. To date, little attention has been devoted to the dosage and the length of treatment, in order to avoid counterproductive effects on the hormonal level such as androgen increase [38, 39, 69], which is especially harmful for PCOS patients. Currently, many authors agree that treatment with the sole DCI is inappropriate in such cases. On the contrary, DCI down-regulation activity on aromatase could be an interesting therapeutic opportunity in males, where T accumulation within certain limits is beneficial. Indeed, as reported in several studies, different ratios among sex hormones characterize male and female sexual spheres [70]. Estrogens, androgens and aromatase expression are fundamental in men, just like in women. Some authors reported the importance of low levels of estrogens in men, particularly for modulating libido, erectile function, and spermatogenesis [71]. In this regard, a correct T/E2 ratio should be equal to or greater than 10. Abnormal T/E2 (< 10) is associated with decreased semen parameters, and administration of an aromatase inhibitor proved to normalize such alterations, yielding to improved sperm concentration, motility, and morphology [71].

Considering its effects on sex hormone balance and its safety profile [42–48, 63], DCI can cover new therapeutic opportunities. DCI may be administered to male patients with “mild hypogonadism”, characterized by low levels of T due to conditions such as hypogonadal and senile hypogonadism, both sexual dysfunctions that need androgen levels to be rebalanced [40, 41].

As previously pointed out, although our results are obtained in males, they can provide suggestions also for therapies in females. Indeed, DCI should be tested in the treatment of hyper-estrogenic conditions, such as endometriosis, and breast and endometrial cancer [40, 41]. Furthermore, administration of DCI, either alone or in combination with aromatase inhibitors (e.g., letrozole, anastrozole, or exemestane) to decrease their dosage, might induce ovulation in some kind of patients [40, 41]. In any case, the length of DCI treatment, as well as its dosage needs to be carefully evaluated for each pathology to avoid unintended effects.

## Conclusions

The preliminary results presented in this report demonstrate, for the first time, that a daily oral administration of 1 g DCI can improve the sexual hormone profile in adult men. As result of DCI treatment, E1 significantly and E2 not significantly decreased, whereas T, DHEAS and Epia increased, resulting in increased T/E2 ratio. The most dramatic change was the significant reduction of E1 serum levels, which may relate to a the direct hypoglycemic activity previously observed for DCI. Altogether, these findings lead the authors to propose that DCI may act as a down-regulator of the aromatase activity. Additional studies with a larger cohort of patients are required to confirm our findings, but also to evaluate the effects on prostate and sperm parameters and to understand the underlying molecular mechanisms involved in DCI activities. A relevant aspect to highlight is the safety profile of DCI, which represents a great advantage with respect to aromatase inhibitors.

The complex activities exerted by DCI offer promising opportunities. DCI may be administered at different dosages and length of treatment to treat in men mood disorders or reduced libido associated to low testosterone levels, hypogonadotropic hypogonadism, overweight or obesity.

## Data Availability

The data generated or analysed during this study are included in this published article. Further information can be obtained from the Corresponding Author.

## Abbreviations

A4: Androstenedione
AIs: Aromatase inhibitors
AUC: Area under the curve
BMI: Body mass index
Cmax: Maximum concentration recorded
CYP19A1: Aromatase gene
DCI: D-chiro-inositol
DHEAS: Dehydroepiandrosterone sulfate
E2: Estradiol
E1: Estrone
ELISA: Enzyme-linked immunosorbent assay
Epia: Epiandrosterone
FSH: Follicle-stimulating hormone
FOXO1: Forkhead transcription factor 1
HOMA: Homeostatic Model Assessment
IPGs: Inositol phosphoglycans
IR: Insulin resistance
LH: Luteinizing hormone
MI: Myo-inositol
PI3K: Phosphatidylinositol 3-kinase
PCOS: Polycystic Ovary Syndrome
AKT: Protein kinase B
t0: Baseline time
t1: Post-DCI treatment time
T: Testosterone
T2DM: Type 2 Diabetes Mellitus
Tmax: Time required to reach Cmax
V1-V10: Volunteers 1 to 10
